# The effects of prefrontal tDCS and hf-tRNS on the processing of positive and negative emotions evoked by video clips in first- and third-person

**DOI:** 10.1038/s41598-024-58702-7

**Published:** 2024-04-05

**Authors:** Pasquale La Malva, Adolfo Di Crosta, Giulia Prete, Irene Ceccato, Matteo Gatti, Eleonora D’Intino, Luca Tommasi, Nicola Mammarella, Rocco Palumbo, Alberto Di Domenico

**Affiliations:** Department of Psychological, Health and Territorial Sciences, “G. d’Annunzio” University of Chieti-Pescara, 31, Via dei Vestini, 66100 Chieti, Italy

**Keywords:** Transcranial direct current stimulation (tDCS), Transcranial random noise stimulation (tRNS), Emotional video, Point of view (POV), Dorsolateral prefrontal cortex (DLPFC), Perception, Neurophysiology

## Abstract

The causal role of the cerebral hemispheres in positive and negative emotion processing remains uncertain. The Right Hemisphere Hypothesis proposes right hemispheric superiority for all emotions, while the Valence Hypothesis suggests the left/right hemisphere's primary involvement in positive/negative emotions, respectively. To address this, emotional video clips were presented during dorsolateral prefrontal cortex (DLPFC) electrical stimulation, incorporating a comparison of tDCS and high frequency tRNS stimulation techniques and manipulating perspective-taking (first-person vs third-person Point of View, POV). Four stimulation conditions were applied while participants were asked to rate emotional video valence: anodal/cathodal tDCS to the left/right DLPFC, reverse configuration (anodal/cathodal on the right/left DLPFC), bilateral hf-tRNS, and sham (control condition). Results revealed significant interactions between stimulation setup, emotional valence, and POV, implicating the DLPFC in emotions and perspective-taking. The right hemisphere played a crucial role in both positive and negative valence, supporting the Right Hemisphere Hypothesis. However, the complex interactions between the brain hemispheres and valence also supported the Valence Hypothesis. Both stimulation techniques (tDCS and tRNS) significantly modulated results. These findings support both hypotheses regarding hemispheric involvement in emotions, underscore the utility of video stimuli, and emphasize the importance of perspective-taking in this field, which is often overlooked.

## Introduction

### Cerebral basis of emotion detection

Facial expressions are crucial for social interactions^1^ and the ability to correctly interpret them is needed to interact proficiently with each other^2^. Emotions are a complex process involving different dimensions^3–5^, including recognition of emotional stimuli, valence, and intensity^6,7^. The cerebral basis of facial emotion detection has been widely investigated using both behavioral paradigms^8–12^, electrophysiological studies^13–15^, and neuroimaging tools^16,17^. It is well-established that emotional contents are simultaneously processed by a rapid, cortical route, and by a slow, subcortical route^18^. Both routes comprise the amygdala; then the subcortical route activates limbic structures, while the cortical route terminates in the occipital-temporal face areas. Importantly, the outcome of both routes is modulated by the activity of the prefrontal areas, with a core role of the dorsolateral prefrontal cortex (DLPFC). In this frame, the role of each hemisphere in processing the valence of emotional stimuli is still debated^8^. Indeed, the reference literature demonstrates how over the years, various hypotheses have been explored (for further details, see “negative only valence hypothesis”^19^ and “modified valence hypothesis”^86^). However, the majority of studies have primarily been based on the following two hypotheses: according to the Right Hemisphere Hypothesis (RHH), the right hemisphere is specialized in the processing of all emotional valences^20^, whereas according to the Valence Hypothesis (VH), the left/right hemispheres are specialized in processing positive/negative emotions, respectively^21^. On the one hand, studies supporting RHH showed that the right DLPFC is predominantly involved in emotion-related working memory tasks^22^ and emotional face perception^23^. On the other hand, studies supporting the VH revealed an association between the activity of the left DLPFC and the processing of positive stimuli and positive mood^24,25^, as well as the activity of the right DLPFC with the processing of negative stimuli and negative mood^16,26,27^. Furthermore, in accordance with VH, clinical depression seems to be associated with left DLPFC hypoactivity and with right DLPFC hyperactivity^28^ and lesions of the left prefrontal cortex due to stroke, tumors, or epilepsy are often accompanied by depression, whereas lesions on the right prefrontal cortex have been related with elated mood^29,30^. Based on these multiple findings, it has been argued that activation of the left DLPFC might reflect alterations of mood and emotions in more positive states^31^, even if a final consensus on the role of each half of the brain in (facial) emotion processing is far to be obtained (see for instance^8,32^).

### Video stimuli and perspective-taking in emotion processing

Most of the evidence concerning the perception of emotional stimuli is collected by using static stimuli. Nevertheless, several studies highlighted that the use of emotional videos can offer additional advantages in the study of emotions in the laboratory^33–36^. Indeed, emotional videos seem to be more effective in eliciting emotions for longer periods at both the subjective and physiological levels, compared to static visual stimuli (e.g., words, pictures^37,38^, facial expressions^39^). Therefore, emotional videos can be used to increase the intensity of the subjective experience in the perception of the valence associated with emotional stimuli^40,41^. Databases constituted by emotional videos have been proposed only recently in the literature, with the specific aim of studying emotion perception. Among these, the Chieti Affective Action Videos (CAAV^42^) is a video database specifically developed for experimental research and its critical feature is perspective-taking since each video is shown both in the third-person and first-person Point of View (POV). Perspective-taking refers to a complex socio-cognitive process that involves the recognition and appreciation of another person’s point of view, which can be the same or different from our own^43,44^. First-person POV, in which individuals observe a scene from their perspective, improves imitative behavior and induces greater activity in the mirror neuron system compared to third-person POV^45,46^. It has also been shown that first-person POV can influence emotional responses while playing video games: first-person playing view generates a greater emotional response and a higher sense of presence in the scene (i.e. immersive) compared to the third-person playing view^47,48^. Therefore, first-person emotional videos would further increase the subjective perception of valence compared to third-person videos.

The activity of DLPFC also plays a role in perspective-taking, which is highly related to human empathy, namely the ability to internally simulate and adopt the mental states of others^49^. Some evidence suggests that right DLPFC activation is related to strong inhibition of own’s egocentric perspective^50^; for instance, the right DLPFC is more active when children take a third-person perspective compared to a first-person perspective^51^ and it has been suggested that the right DLPFC may be strongly involved in children because they required greater inhibition to suppress their egocentric perspective. Right DLPFC is also associated with the inhibition of egocentric perspective in decision-making tasks based on self-interest^52,53^.

In conclusion, through different types of tasks and procedures, DLPFC is related to monitoring processes involved in the recognition and judgment of one's own personal versus another's emotional state. However, results on the specific contributions of each hemisphere in perspective-taking are controversial. Furthermore, many studies are focused on the role of DLPFC in perspective-taking with cognitive tasks, but the possible interactions with emotion recognition and emotional processing are poorly investigated. As a matter of fact, the role of different POVs in influencing the subjective perception of emotional valence and the specific contribution of DLPFC in these interaction processes still represents an unexplored issue.

### Transcranial electrical stimulation

A non-invasive tool used to shed more light on the cerebral basis of emotion perception is transcranial electrical stimulation (tES). It includes a series of stimulation techniques that have been widely exploited in the last decades to investigate the causal relationship between cortical activity and cognitive/perceptual tasks^54,55^. Among these, the most exploited is transcranial Direct Current Stimulation (tDCS), in which the cortical excitability is modulated in a polarity-dependent way: the current flows from one electrode to another one, inducing a polarization of cortical neurons at a subthreshold level^56^. The effects of tDCS can be positive or negative, meaning that “anodal stimulation” induces a cellular membrane depolarization (positive effect) in the target area (facilitation of neural processing), whereas cathodal stimulation induces a hyperpolarization (negative effect) in the target area (inhibition of neural processing^57–60^). In head models, the median current density tends to decrease with increasing distance from the electrodes, even if a certain spatial resolution can be obtained^61^.

Transcranial electrical current can be also delivered by exploiting the application of repetitive alternating, instead of direct, current over the cortex at random frequencies, namely transcranial Random Noise Stimulation (tRNS). It can be delivered from 0.1 to 640 Hz, or it can be used only at low (0.1–100 Hz) or only at high (100–640 Hz) frequency. It has been found that the application of high-frequency tRNS (hf-tRNS) positively modulates the excitability of motor and perceptual areas^62,63^, and also improves performance in behavioral tasks, for example in the domain of motor and visual perception learning^64,65^. To summarize, tES modulates cortical activity and in the present study, we exploited two different types of stimulation to investigate whether and in which direction these techniques could be exploited to enhance emotion recognition expressed by emotional videos with different POVs.

### The present study

As reviewed above, the DLPFC plays a key role in emotional processing and perspective-taking, especially in the subjective perception of valence associated with an emotional stimulus^6,17,66^. A mole of neuroimaging evidence highlighted that DLPFC is involved in emotion-related brain networks, through connections with the amygdala and other subcortical nuclei^67–69^. Studies exploring the effects of tES in emotional processing in healthy participants showed mixed results: Vierheilig and colleagues^70^ found that tDCS applied bilaterally on the prefrontal cortex did not influence emotional processing. However, promising results have been obtained by applying anodal tDCS on left DLPFC, which led to a decrease in the perception of the emotional value of unpleasant pictures^71,72^. By using pictures from the International Affective Pictures System (IAPS^73^), a study^74^ revealed that negative stimuli were evaluated as less negative after anodal tDCS over left DLPFC (through an intensity of 1 mA with anode on F3 and cathode on C4) compared to both cathodal tDCS and sham (control) conditions. Moreover, concerning emotional face identification, anodal tDCS over left DLPFC (on F3) improved emotional processing for positive stimuli compared to negative stimuli^31,75^, supporting the VH. Finally, partially contradictory results were collected also according to perspective-taking: anodal right/cathodal left tDCS applied over the DLPFC (through an intensity of 1 mA with anode on F4 and cathode on F3) negatively influenced participants’ ability to adopt another’s POV in a visual perspective-taking task^76^. However, subjective ratings for others’ pain increased with anodal tDCS on the left DLPFC^77^ compared to sham, indicating enhanced pain empathy.

We can conclude that anodal tDCS applied on the DLPFC, especially on the left hemisphere, seems to influence emotional processing in healthy individuals, leading to perceiving negative stimuli as less negative and improving the identification of positive stimuli. These results on the specific role of the left DLPFC partially support the VH, but also different evidence has been described^23,78^. Furthermore, to fully confirm the VH in a tDCS protocol, then not only facilitatory (anodal) stimulation on the left DLPFC should selectively enhance emotional processing for positive stimuli (as shown in the studies mentioned above), but also stimulation on the homolog right area should selectively enhance emotional processing for negative stimuli^75^. Since this clear pattern has not been confirmed yet, the frame of hemispheric asymmetries in emotion detection and perspective-taking is still open.

Concerning tRNS, the absence of a clearcut current polarity leads to often use of this technique by using a bilateral cephalic montage (left and right DLPFC stimulated simultaneously). In addition, unlike tDCS, the tRNS technique has intrinsic temporal variability of stimulation parameters, which may be more effective in interfering with rapid neural mechanisms^82^. Also in this case, results are not clear (and they are also relatively scarce) in the domain of emotion processing: for instance, bilateral hf-tRNS leads to a positive mood change^79^, but it was inefficient in reducing the symptomatology in a sample of depressed patients^80^. At a mere perceptual level, a study targeting either the left or the right DLPFC showed no effects of hf-tRNS in the judgment of the friendliness level of photographs showing emotional faces^81^.

The present study aims to disentangle the issue of the contribution of the DLPFC in emotional processing and perspective-taking, focusing on the effects of i) left/right tDCS and ii) hf-tRNS applied bilaterally on DLPFC, on the perception of emotional videos presented with different POV. In a between-subjects design, we defined four experimental groups according to stimulation setup: Left Anodal/Right Cathodal tDCS (tDCS-LA/RC); Right Anodal/Left Cathodal tDCS (tDCS-RA/LC); hf-tRNS (bilateral stimulation), and a sham control condition. We hypothesized that, following the VH, during left/right anodal tDCS the valence of emotional stimuli could be perceived as more positive/negative, respectively, compared to sham condition. Moreover, we also hypothesized that hf-tRNS modulates neuronal activity more than tDCS, as found in some fields different from emotions^64,82^, independently of hemispheric specialization possibly due to the importance of inter-hemispheric exchanges in correctly decoding both positive and negative emotions. Furthermore, a fundamental aspect of the present study consists in the specific set of stimuli used: to the best of our knowledge, this is the first study that used video stimuli to explore tES effects on emotional processing (positive results have been very recently described by applying tRNS on the cerebellum^62^). The identified database also provides videos with neutral emotional valence, which were used in the study as control stimuli for those with positive and negative valence. Additionally, these stimuli allow us to explore the further effects of perspective-taking (first-person POV vs third-person POV) on the perception of emotional valence. We hypothesized that: (i) first-person POV would induce stronger emotional evaluation than third-person POV for all stimuli; (ii) anodal tDCS over the left/right DLPFC would enhance the positive/negative emotional evaluation, respectively, following the VH; (iii) hf-tRNS would lead to even stronger effects than tDCS.

## Results

Statistical analyses were performed using Statistica 8.0 software (StatSoft. Inc., Tulsa, USA). A 4 × 3 × 2 repeated-measures Analysis of Variance (ANOVA) was carried out with Stimulation (tDCS-LA/RC, tDCS-RA/LC, tRNS, Sham) as a between-subjects factor, and with Valence (Positive, Neutral, Negative) and POV (first-person POV: 1-POV, third-person POV: 3-POV) as within-subjects factors. The participants’ ratings on the SAM valence scale were entered as the dependent variable. Post-hoc analyses were performed using Tukey HSD tests and the significance level was set at *p* < 0.05.

**Figure 1:**
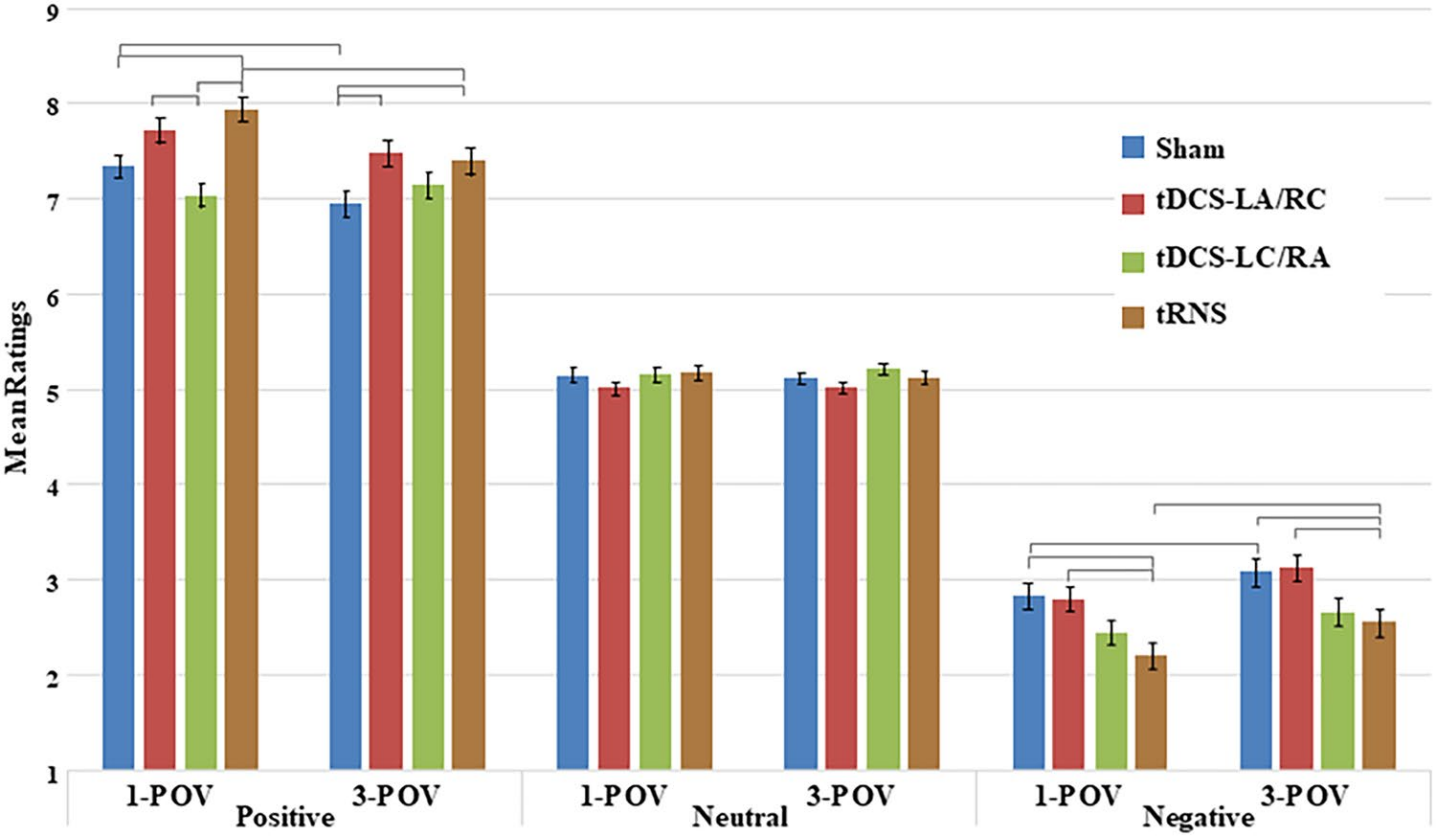
Interaction Stimulation, Valence, and POV on participants’ valence ratings*.* Error Bars represent Standard Errors. Brackets identify statistically significant differences involving POV and Stimulation. *P* values are reported in Table 1.

The three-way interaction among Stimulation, Valence, and POV was also significant, *F*(6, 184) = 3.09, *p* < 0.01, $\eta_{p}^{2}$ = 0.09 (see Fig. 1), and post-hoc comparisons (see Table 1) confirmed that, regardless of Stimulation and POV, ratings were higher for Positive and lower for Negative stimuli compared to Neutral ones, all *ps* < 0.001. Focusing on the Positive stimuli shown in the 1-POV, the ratings in the tRNS were higher compared to Sham (*p* < 0.001), but no difference emerged between Sham and either tDCS-RA/LC (*p* = 0.66) and tDCS-LA/RC (*p* = 0.26). For Positive valence and 3-POV, ratings were higher in the tDCS-LA/RC (*p* < 0.01) and in the tRNS (*p* < 0.05) compared to Sham, whereas no difference emerged for tDCS-RA/LC compared to Sham (*p* = 0.99). Finally, ratings for 1-POV were higher compared to 3-POV both in Sham (*p* < 0.01) and in tRNS (*p* < 0.001). Focusing on the Negative valence, 1-POV ratings in the tDCS-RA/LC (*p* < 0.001) and in the tRNS (*p* < 0.001) were lower compared to the Sham condition; instead for 3-POV ratings were lower in the tRNS (*p* < 0.001) compared to Sham. Analyzing the POV within-subjects factor, ratings for 1-POV were lower compared to 3-POV videos both in the Sham (*p* < 0.001) and in the tRNS (*p* < 0.05). Finally, focusing on the Neutral valence, no difference emerged.

**Table 1:** Means, Standard Errors, and Pairwise comparisons (p-values for Tukey HSD) for the repeated measures three-way ANOVA on participants’ ratings.

	Sham Pos-1	Neu-1	Sham Neg-1	LC/RA Pos-1	LC/RA Neu-1	LC/RA Neg-1	LA/RC Pos-1	LA/RC Neu-1	LA/RC Neg-1	tRNS Pos-1	tRNS Neu-1	tRNS Neg-1	Sham Pos-3	Sham Neu-3	Sham Neg-3	LC/RA Pos-3	LC/RA Neu-3	LC/RA Neg-3	LA/RC Pos-3	LA/RC Neu-3	LA/RC Neg-3	tRNS Pos-3	tRNS Neu-3	tRNS Neg-3
M	7.34	5.15	2.84	7.04	5.16	2.45	7.71	5.01	2.80	7.93	5.18	2.20	6.95	5.11	3.09	7.14	5.21	2.66	7.48	5.02	3.13	7.39	5.12	2.56
*SE*	0.13	0.07	0.13	0.13	0.07	0.13	0.13	0.07	0.13	0.13	0.07	0.13	0.14	0.07	0.15	0.14	0.07	0.15	0.14	0.07	0.15	0.14	0.07	0.15
Sham Pos-1		< 0.001	< 0.001	0.657	< 0.001	< 0.001	0.261	< 0.001	< 0.001	< 0.001	< 0.001	< 0.001	0.007	< 0.001	< 0.001	0.993	< 0.001	< 0.001	0.999	< 0.001	< 0.001	0.999	< 0.001	< 0.001
Sham Neu-1			< 0.001	< 0.001	0.999	< 0.001	< 0.001	0.999	< 0.001	< 0.001	0.999	< 0.001	< 0.001	0.999	< 0.001	< 0.001	0.999	< 0.001	< 0.001	0.999	< 0.001	< 0.001	0.999	< 0.001
Sham Neg-1				< 0.001	< 0.001	0.183	< 0.001	< 0.001	0.999	< 0.001	< 0.001	< 0.001	< 0.001	< 0.001	0.581	< 0.001	< 0.001	0.999	< 0.001	< 0.001	0.745	< 0.001	< 0.001	0.810
LC/RA Pos-1					< 0.001	< 0.001	< 0.001	< 0.001	< 0.001	< 0.001	< 0.001	< 0.001	0.999	< 0.001	< 0.001	0.999	< 0.001	< 0.001	0.051	< 0.001	< 0.001	0.328	< 0.001	< 0.001
LC/RA Neu-1						< 0.001	< 0.001	0.999	< 0.001	< 0.001	0.999	< 0.001	< 0.001	0.999	< 0.001	< 0.001	0.999	< 0.001	< 0.001	0.999	< 0.001	< 0.001	0.999	< 0.001
LC/RA Neg-1							< 0.001	< 0.001	0.356	< 0.001	< 0.001	0.946	< 0.001	< 0.001	< 0.001	< 0.001	< 0.001	0.880	< 0.001	< 0.001	< 0.001	< 0.001	< 0.001	0.999
LA/RC Pos-1								< 0.001	< 0.001	0.985	< 0.001	< 0.001	< 0.001	< 0.001	< 0.001	0.001	< 0.001	< 0.001	0.693	< 0.001	< 0.001	0.573	< 0.001	< 0.001
LA/RC Neu-1									< 0.001	< 0.001	0.999	< 0.001	< 0.001	0.999	< 0.001	< 0.001	0.996	< 0.001	< 0.001	0.999	< 0.001	< 0.001	0.999	< 0.001
LA/RC Neg-1										< 0.001	< 0.001	< 0.001	< 0.001	< 0.001	0.784	< 0.001	< 0.001	0.999	< 0.001	< 0.001	0.096	< 0.001	< 0.001	0.942
tRNS Pos-1											< 0.001	< 0.001	< 0.001	< 0.001	< 0.001	< 0.001	< 0.001	< 0.001	0.036	< 0.001	< 0.001	< 0.001	< 0.001	< 0.001
tRNS Neu-1												< 0.001	< 0.001	0.999	< 0.001	< 0.001	0.999	< 0.001	< 0.001	0.999	< 0.001	< 0.001	0.999	< 0.001
tRNS Neg-1													< 0.001	< 0.001	< 0.001	< 0.001	< 0.001	0.038	< 0.001	< 0.001	< 0.001	< 0.001	< 0.001	0.043
Sham Pos-3														< 0.001	< 0.001	0.997	< 0.001	< 0.001	0.003	< 0.001	< 0.001	0.042	< 0.001	< 0.001
Sham Neu-3															< 0.001	< 0.001	0.999	< 0.001	< 0.001	0.999	< 0.001	< 0.001	0.999	< 0.001
Sham Neg-3																< 0.001	< 0.001	0.068	< 0.001	< 0.001	0.999	< 0.001	< 0.001	0.003
LC/RA Pos-3																	< 0.001	< 0.001	0.441	< 0.001	< 0.001	0.909	< 0.001	< 0.001
LC/RA Neu-3																		< 0.001	< 0.001	0.998	< 0.001	< 0.001	0.999	< 0.001
LC/RA Neg-3																			< 0.001	< 0.001	0.020	< 0.001	< 0.001	0.999
LA/RC Pos-3																				< 0.001	< 0.001	0.999	< 0.001	< 0.001
LA/RC Neu-3																					< 0.001	< 0.001	0.999	< 0.001
LA/RC Neg-3																						< 0.001	< 0.001	0.001
tRNS Pos-3																							< 0.001	< 0.001
tRNS Neg-3																								< 0.001

LC/RA = tDCS-LC/RA; LA/RC = tDCS-LA/RC; Pos = Positive; Neu = Neutral; Neg = Negative; 1 = POV-1; 3 = POV-3.

The interaction between Stimulation and Valence was significant, *F*(6, 184) = 5.18, *p* < 0.001, $\eta_{p}^{2}$ = 0.14, as well as the interaction between Stimulation and POV, *F*(3, 92) = 2.90, *p* < 0.05, $\eta_{p}^{2}$ = 0.11. The main effect of Stimulation was significant, *F*(3, 92) = 4.86, *p* < 0.01, $\eta_{p}^{2}$ = 0.14, with a difference between the two tDCS conditions, showing lower ratings in the tDCS-RA/LC than in the tDCS-LA/RC condition, *p* = 0.01. The main effect of Valence was significant, *F*(2, 184) = 1492.82, *p* < 0.01, $\eta_{p}^{2}$ = 0.94, with post-hoc indicated that ratings were higher for Positive stimuli and lower for Negative stimuli compared to Neutral ones, all *ps* < 0.001. Finally, the main effect of POV was not significant, *F*(1, 92) = 0.01, *p* = 0.93, $\eta_{p}^{2}$ = 0.00, but it significantly interacted with Valence, *F*(2, 184) = 33.39, *p* < 0.001, $\eta_{p}^{2}$ = 0.25. Post-hoc showed that for Positive stimuli, 1-POV obtained higher ratings compared to 3-POV, whereas for Negative stimuli 1-POV obtained lower ratings compared to 3-POV, all *ps* < 0.001. No statistical difference emerged for the Neutral valence, *p* = 0.999.

## Discussion

The present study aimed to assess the causal role of DLPFC on the valence ratings of emotional videos, in accordance with perspective-taking. In particular, we exploited electrical stimulation to investigate this issue, starting from three specific hypotheses: (i) first-person POV leads to stronger emotional evaluation than third-person POV, due to a facilitation in perspective-taking when videos are presented in first-person^45–48^. Moreover, (ii) according to the VH^11,83,84^, we hypothesized the hyper/hypo-activity of the left/right hemisphere (tDCS-LA/RC over the DLPFC) respectively should enhance the positive emotional evaluation as opposed to the hypo/hyper-activity of the left/right hemisphere (tDCS-RA/LC over the DLPFC) respectively, which should enhance the negative emotional evaluation, thus confirming a crucial role of the left hemisphere in positive valence and that of the right hemisphere in negative valence. Finally, starting from previous evidence, we also expected that (iii) hf-tRNS applied bilaterally on the DLPFC should lead to even more extreme emotional ratings than tDCS, for both positive and negative valence^64,82^.

The results of the presented study partially support our hypotheses. Firstly, both the main effect of Valence and all its interactions confirmed that participants’ ratings were higher for positive and lower for negative stimuli with respect to neutral videos, thus ensuring the video database is a valid tool for studying emotional response. Concerning the first hypothesis, the main effect of POV was not significant, thus revealing no difference in absolute emotional ratings according to perspective-taking. However, an interesting finding emerges as an interaction between POV and valence, revealing that valence ratings were more positive for positive stimuli presented in the first- than in third-person, but they were more negative for negative stimuli presented in the first- than in third-person, with no difference for neutral stimuli. This interaction highlights a link between emotional valence and perspective-taking (regardless of prefrontal stimulation) which confirms that first-person videos make emotional valence more salient than third-person videos, and thus supporting the evidence found during playing videogames, showing first-person POV to generate greater immersivity and thus emotional responses than third-person POV^47,48^. This result is crucial in the literature on emotional valence because it highlights the importance of perspective-taking in studying the processing of positive and negative stimuli, a topic that received little attention in this literature and needs to be further explored. Importantly, no effects of stimulation emerged for perspective-taking by using emotional videos.

Concerning the second hypothesis, the difference among groups showed an unexpected result: differently from the starting hypothesis, no difference emerged between the two tDCS groups, preventing us from clearly supporting the VH. Contrary to our expectation, results showed higher valence ratings when the right DLPFC activity was enhanced by tDCS (independently of the valence of the videos) compared to an enhanced left DLPFC activity, thus revealing a crucial role of the right hemisphere in all emotion processing, as predicted by the RHH^20,85^. In fact, emotional ratings were higher in the group in which tDCS was applied with right-anodal and left-cathodal montage compared with the group in which it was applied with left-anodal and right-cathodal montage. This finding partially replies also to our last hypothesis, since no difference emerged in the direct comparison between tDCS and tRNS groups, suggesting the absence of the expected stronger effect of tRNS applied on the DLPFC. However, the three-way interaction sheds more light on these findings: it interestingly showed that even if the direct comparison between tDCS and tRNS did not reach significance, valence ratings were higher during tRNS compared to sham when videos with a positive valence were presented both in first- and in third-person POV. In this condition (positive videos) also the ratings recorded during tDCS applied with anodic stimulation on the left DLPFC were higher with respect to sham, but only for third-person POV. On the contrary, valence ratings were lower during tRNS compared to sham when videos with a negative valence were presented both in first- and in third-person, and in this condition (negative videos) also the ratings recorded during tDCS applied with anodic stimulation on the right DLPFC were lower with respect to sham, again only for third-person POV. This peculiar pattern of results suggests that when first-person perspective is adopted, the emotional evaluation is strong enough to be not modulated by lateralized tDCS, however when emotional videos are shown in third-person, emotional ratings are influenced not only by tRNS but also by tDCS: the enhancement of the activity of the left DLPFC leads participants to express more positive ratings for positive valence stimuli, but the enhancement of the activity of the right DLPFC leads participants to express more negative ratings for negative valence stimuli. This pattern is exactly in line with the VH, showing a determinant role of the left/right hemisphere for positive/negative stimuli respectively. Even if this conclusion seems to be in contrast with the support for the RHH described above starting from the main effect of stimulation, it nevertheless remarks previous findings highlighting alternating support for each of the two main hypotheses on the role of each cerebral hemisphere in emotion processing^8,86^. It has been proposed that the two hypotheses are not mutually exclusive, but that they can be both valid and supported depending on the specific task required: in this case, we can speculate that the third-person POV, which is less immersive than the first-person POV, would highlight a hemispheric division of emotional processing, possibly due to the higher cognitive demand required to process this kind of emotional videos. This speculation needs to be further explored because the paucity of research investigating the relationship between valence and perspective-taking prevents us from being conclusive on this point.

Finally, the present results also showed that when videos contain positive valence, they received higher (more positive) ratings when the first POV was presented compared to the third POV, both in the control group (sham) and the tRNS group, whereas the hemispheric imbalance due to lateralized tDCS did not lead to a significant difference. The exact opposite pattern emerges with negative valence stimuli, which received lower (more negative) ratings when the first POV was presented compared with the third POV, again in the control group (sham) and tRNS, with no difference in the tDCS groups. These findings revealed that the hemispheric imbalance due to lateralized tDCS influences the expected superiority of first-person perspective-taking, which remains stable when both hemispheres are stimulated by tRNS. Importantly, no difference emerged when the videos showed neutral valence actions.

To conclude, this study shows the importance of considering perspective-taking in the study of emotional valence. It confirms that emotional videos are a valid tool to investigate this issue and it also provides further support for the involvement of the DLPFC in this domain. Further studies are needed to clarify the interaction among emotional valence, perspective-taking and prefrontal hemispheric involvement. Indeed, some limitations of this study could be addressed through the use of multiple instruments or innovative tools for electrophysiological detection (e.g., EEG) that allow the acquisition of neuronal electrical activity data simultaneously with the use of tES techniques. Specifically, such measurements would enable the control of possible individual differences in neurological activation. Moreover, the stimulation procedure could be replicated by employing a unilateral electrode montage (placing the second electrode on an extracerebral area) to investigate more specifically the effects of stimulation on only one hemisphere. Further studies should also consider the levels of arousal elicited by emotional stimuli in order not to overlook possible implications. However, the present results suggest that both most supported theories on hemispheric processing of positive and negative valence are accurate, with the main effect of stimulation supporting the RHH and the three-way interaction supporting the VH, at least for third-person stimuli. Finally, the stronger involvement in first- compared to third-person emotional stimuli is confirmed, with a higher modulation of the emotional ratings induced by bilateral tRNS than by lateralized tDCS, suggesting that perspective-taking requires the activity of both hemispheres.

## Material and methods

### Participants

Twenty-four participants for each stimulation condition, for a total of ninety-six participants, (50% female) between 18 and 30 years old (M = 23.99; SD = 3.45) took part in the study. All participants were right-handers (M = 61.23; SD = 20.19) as measured using the Edinburgh Handedness Inventory^87^. Participants reported normal or corrected-to-normal vision and were free from a medical history of psychiatric or neurological conditions. Before the task, each participant completed the handedness inventory, and both BDI-II^88^ to quantify depression scores (M = 7.46; SD = 3.43; all participants had a score lower than 13, which is the cutoff indicating the presence of depressed mood), and PANAS^89^, to quantify positive (M = 31.03; SD = 6.83) and negative (M = 21.77; SD = 6.42) affect scores. The study was single-blind and each participant was randomly assigned to one of the four experimental groups, with gender balance ensured. One-way ANOVA revealed no difference among groups concerning age, BDI-II, positive PANAS, and negative PANAS scores (see Table 2). The study was carried out in accordance with the Declaration of Helsinki and was approved by the Ethical Committee of the Department of Psychological, Health and Territorial Sciences, University “G. d’Annunzio”. Each participant provided written informed consent before beginning the task and none reported discomfort or distress after completing the experiment.

**Table 2:** Means and Standard Deviations of variables included in the present study as a function of experimental groups based on stimulation conditions.

Variable	Sham		tDCS-RA-LC		tDCS-LA-RC		tRNS		One-way ANOVA
	*M*	*SD*	*M*	*SD*	*M*	*SD*	*M*	*SD*	
Age	24.33	3.17	23.17	3.69	23.17	3.32	25.29	3.30	*F*(3,95) = 2.22, *p* = 0.09, $\eta_{p}^{2}$ = 0.07
Panas+	32.38	5.32	30.08	6.76	30.83	6.53	32.25	6.75	*F*(3,95) = 0.74, *p* = 0.53, $\eta_{p}^{2}$ = 0.02
Panas−	21.83	5.79	21.54	6.49	22.71	7.65	21.00	5.88	*F*(3,95) = 0.29, *p* = 0.83, $\eta_{p}^{2}$ = 0.01
BDI-II	8.33	3.92	7.00	3.16	6.58	3.24	7.92	3.26	*F*(3,95) = 1.34, *p* = 0.27, $\eta_{p}^{2}$ = 0.04

### Stimuli

Video extracted from the CAAV database^42,90^ were used as stimuli: CAAV consists of 360 videos with different emotional valence (negative, neutral, positive), including 90 different actions lasting 15 s and recorded without sound. Each action is filmed in four different versions (for a total of 360 stimuli) based on the gender of the main actor and on the perspective in which each action was recorded: (1) first-person POV, female actor; (2) first-person POV, male actor; (3) third-person POV, female actor; (4) third-person POV, male actor. CAAV ratings were originally obtained using the Self-Assessment Manikin (SAM) scale^91^ ranging from 1 (very negative valence) to 9 (very positive valence). In the present study we included 30 negative actions (e.g., “coughing blood”), each reporting a mean valence between 2.27 and 3.85 (M = 2.93; SD = 0.46), 30 neutral actions (e.g., “finger counting”), each reporting a mean valence between 4.45 and 5.54 (M = 5.05; SD = 0.26), and 30 positive actions (e.g., “eating cake”), each reporting a mean valence between 5.87 and 7.49 (M = 6.56; SD = 0.41). We created four experimental lists (A, B, C, D) balancing their administration among experimental groups: each list included all 90 actions, which were shown in one of the four versions varying perspective and gender of the actor (e.g., the action “coughing blood” was presented with a female actor and first-person POV in list A, with a male actor and first-person POV in list B, with a female actor and third-person POV in list C, with a male actor and third-person POV in list D). Accordingly, each participant was presented with only one video version of each action.

### Procedure

Participants were invited to sit in a dark and silent room, to sign the informed consent and to complete handedness, BDI-II and PANAS questionnaires. Then the stimulation montage was set, and after 5 min from the onset of the stimulation the computerized task began. Each of the CAAV actions was randomly presented (30 negative, 30 neutral, 30 positive) and participants were asked to rate each stimulus. Each trial started with a central fixation cross (500 ms), followed by a video (15 s) presented in the center of a 15-inch laptop screen. Then, participants were presented with the Self-Assessment Manikin (SAM) scale^91^ ranging from 1 (i.e., very negative valence) to 9 (i.e., very positive valence) and they were instructed to rate the valence of each video by pressing the corresponding key on the laptop keyboard (max response duration: 6 s). The task lasted about 34 min and it was administered via E-Prime 3.0 software (Psychology Software Tools, Inc., Pittsburgh, PA). At the end of the experimental session, a debriefing was conducted with each participant. During the debriefing, the blinding condition was checked, and participants were asked if they perceived any impactful sensations due to stimulation.

### Transcranial electrical stimulation

Transcranial Electrical Stimulation was delivered through a battery-driven, constant current stimulator (DC-Stimulator, NeuroConn GmbH, Germany; distributed by EMS, Italy), using a pair of surface saline-soaked sponge electrodes (3 × 3 cm) kept firm by elastic bands. In each condition, a bilateral stimulation was implemented since the two electrodes were placed over homolog areas of the two cerebral hemispheres (i.e., F3 and F4 of the International 10–20 EEG system). The stimulation was applied with an intensity of 1 mA for 40 min, including a fade-in and a fade-out period of 1 min. In the tDCS-LA/RC, the anode was placed on the F3 site and the cathode on the F4, whereas in the tDCS-RA/LC the two electrodes were inverted. For the tRNS condition, a random noise current was applied at high frequency (100–640 Hz). Both in the tRNS and the sham condition the same electrodes and the same montage as described for tDCS were applied, but the stimulation during the sham lasted only 30 s. In all conditions, the task started 5 min after the beginning of the stimulation and was completed online.

## Data Availability

The data that support the findings of this study are available from the corresponding author on reasonable request.
